# Fertilization fallout and fish fertility in low-silt ponds: studying the impacts on soil properties, water quality, immune-physiological response, and reproductive performance in red tilapia broodstock under saline conditions and plant-based diets

**DOI:** 10.1186/s12917-025-05127-7

**Published:** 2025-11-25

**Authors:** Ghada R. Sallam, Mohamed M. Abdel-Rahim, Ahmed E. Sallam, Mona M. Mourad, Ayman M. Lotfy, Mohamed A. Al-absawey, Hafez A-H Mabrouk, Walied M. Fayed, Hala Saber Khalil, Ashraf. I.G. Elhetawy

**Affiliations:** 1 Aquaculture Division, National Institute of Oceanography and Fisheries, NIOF, Cairo, Egypt; 2 Animal and Fish Production Department, Faculty of Agriculture, Alexandria University, P.O. Box 21531, Saba-basha, Alexandria, Egypt; 3 Aquaculture Department, Faculty of Fish Resources,, Suez University, Suez, 43221 Egypt

**Keywords:** Fertilization, Low-silt soils, High salinity, Red tilapia reproduction, Plant-based diets, Seed production

## Abstract

A 210-day reproduction trial tested the effect of organic fertilizer composed of fish sludge + compost of *Beta vulgaris* leaves (FS + BVL) on water quality, soil properties and fertility of red tilapia *Oreochromis sp*. fed a plant-based diet under varying salinities in low-silt ponds. A 1:3 sex ratio (500♂: 1500♀) was stocked into 32 hapas (24.3 m^3^ each) placed in 8 earthen ponds (150 m² each). Four ponds held 18‰ saltwater (2 fertilized, 2 unfertilized) and four ponds held 36‰ saltwater (2 fertilized, 2 unfertilized). Each fertilized and unfertilized group were fed two diets (fishmeal-enriched diet, FM_1_, and plant-based fishmeal-free diet, FM_0_) at 1% of body weight daily. Eight groups were administered in four replicates. The findings revealed that the use of FS + BVL fertilizer significantly (P *< 0.05*) improved water quality (higher oxygen, lower ammonia/nitrite, more chlorophyll and zooplankton) and enhanced nutritional composition of red tilapia (higher muscle protein, lower lipid). Fertilization also improved reproductive indices (testosomatic and gonadosomatic), reduced stress and metabolic markers (cholesterol, liver and kidney enzymes, cortisol), and boosted immunity, antioxidant defenses, and digestive enzyme activities. Consequently, broodstock in FS + BVL ponds achieved markedly (P *< 0.05*) higher sex hormones, fecundity, fry output, and reproductive success. Overall, FS + BVL proved highly effective in promoting red tilapia health and reproduction across 18‰ and 36‰ salinities and different diet types in low-silt ponds.

## Introduction

Aquaculture has become a major contributor to global food security, producing over 94 million tons (51%) in 2022 and surpassing capture fisheries [1]. Tilapia (*Oreochromis* sp.) ranks fourth among cultured species, with 17 species farmed worldwide [2, 3]. Despite this growth, the sector faces key challenges, including freshwater scarcity due to competing agricultural and domestic demands [4], and high feed costs, which represent 60–80% of total fish farming expenses [5, 6]. These pressures have encouraged the use of plant waste and the practice of farming in marginal environments, though such approaches often compromise fish health and productivity [5].

In conventional aquaculture systems, clay-rich soils are valued for their ability to retain water and nutrients, thereby fostering microbial activity and aquatic plant growth [7, 8]. However, the expansion of global aquaculture has increasingly moved into sandy and low-silt soils, which lack these beneficial properties and consequently limit pond productivity and fish performance [7, 9]. To address these limitations, several strategies have been investigated, including the use of clay or synthetic liners such as geotextiles (e.g., HDPE) to minimize seepage [10], recirculating aquaculture systems (RAS) that bypass soil dependence [11], and the application of organic matter from manure, plant residues, or aquaculture sludge to enhance soil fertility and water retention [12, 13]. Recently, marine-derived organic fertilizers have also been proposed as innovative tools to rehabilitate degraded soils while reducing waste from marine sources [14].

Organic fertilizers not only enrich pond soils but also improve water quality, fish performance, and overall aquaculture output [15, 16]. By supplying essential nutrients such as nitrogen, phosphorus, and potassium, they stimulate the growth of phytoplankton and zooplankton, which form the base of the pond food web [17]. For instance, cow manure has been shown to enhance bacterial communities and plankton biomass, thereby supporting higher fish yields [13, 18]. Likewise, aquaculture sludge, a by-product consisting of feces and uneaten feed and sometimes representing up to 35% of feed inputs, serves as a sustainable fertilizer source that reduces environmental waste [12, 19]. When combined with plant residues, sludge contributes to a balanced nutrient profile that promotes favorable pond conditions and maximizes the efficiency of supplementary feeding [16, 20]. Since phytoplankton act as both a natural food source and a regulator of pond ecosystems [20], the demonstrated value of organic fertilizers in freshwater systems points to an underexplored potential in saline aquaculture, particularly where salinity imposes additional stress on fish reproduction.

Salinity is a critical factor regulating gametogenesis and fertility in fish. Although red tilapia (*Oreochromis spp*.) is euryhaline, their reproductive success remains sensitive to salinity fluctuations [21, 22]. Deviations from optimal salinity increase osmoregulatory energy demands, particularly for maintaining sodium and chloride balance [21, 23], while interfering with steroid hormone pathways that influence immunity, growth, and reproductive physiology [24]. Chronic or fluctuating salinity may further result in cytogenetic damage, embryonic malformations, reduced fertility, and delayed hatching [23, 25]. These challenges are magnified when fish are fed plant-based diets, which often lack the amino acid balance and energy density of fishmeal-based feeds [26]. As highlighted by Chen and Liu [27], both diet quality and salinity tolerance are key determinants of reproductive performance, underscoring the need for strategies that mitigate the combined stresses of saline conditions, plant-based feeding, and poor soil fertility [22, 24].

Red tilapia hybrids are among the most preferred tilapia species for commercial aquaculture due to their morphology and appearance, capacity to thrive in varying salinities from zero to marine waters, rapid growth, adaptability to various farming methods and high marketing prices [4, 28]. However, despite being a salt-tolerant species, studies demonstrate a drop in red tilapia reproduction with increasing water salinity, with the critical point of 18‱ being particularly pronounced [21, 29]. The production of eggs and sperm requires a large energy investment, highlighting the crucial role of balanced nutrition in osmoregulation and successful reproduction [24], and demonstrating that providing the energy requirements of maternal fish is vital [30]. The overlapping pressures of saline water, infertile soils, and reduced fishmeal inclusion highlight the need for alternative inputs that support broodstock fertility. One promising approach involves organic fertilizers derived from fish sludge (FS) and *Beta vulgaris* leaves (BVL). *B. vulgaris* leaves, an abundant agricultural by-product, are rich in bioactive compounds such as antioxidants, phenolic compounds, carotenoids, minerals, and vitamins. Their high β-carotene content, in particular, enhances hematopoiesis, immunity, and red blood cell production [31, 32], Their high β-carotene content, in particular, enhances hematopoiesis, immunity, and red blood cell production [30, 31], while their broader bioactive profile supports digestion, stimulates appetite and gut function, reduces inflammation, and resistance to oxidative stress [31, 33].

Against this background, the present study investigates the influence of using FS + BVL as an organic fertilizer (FR) in low-silt ponds on soil properties, water quality, antioxidant capacity, immunity, sexual maturity, and reproductive performance of red tilapia broodstock fed either plant-based free of fishmeal (FM_0_) or fishmeal-enriched (FM_1_) diets under salinities of 18 and 36‰. It is hypothesized that FS + BVL will provide synergistic benefits that improve pond fertility, physiological resilience, and reproductive outcomes compared to either input alone. The findings are expected to advance sustainable aquaculture practices by demonstrating the potential of integrated fish and plant waste utilization to enhance broodstock performance in saline environments.

## Materials and methods

### Study area and soil analyses

The study was carried out at a private fish farm in Dheraa Al Bahri, Borg El Arab, Alexandria Governorate (Gharbian region, Egypt), where the soil is characterized by low fertility (Table 1). It aimed to determine whether desert soil ponds could support red tilapia (*Oreochromis* sp.) breeding under high-salinity conditions through the application of organic fertilizers. In addition, it evaluated the contribution of fertilization to enhancing natural plankton production and its subsequent effects on the spawning performance of red tilapia broodstock fed a fishmeal-free diet (FM_0_).

**Table 1:** Low-silt soil properties in experimental ponds used for red tilapia reproduction

Item	Value (%)
Organic carbon (C %)	0.32
Total Nitrogen (mg/100 g)	50.75
Available Nitrogen (mg/100 g)	0.40
C/N ratio	6.31
Total phosphorus (mg/100 g)	7.14
Available phosphorus (mg/100 g)	0.39
CaCO3	2.64
pH	6.88
Sand (%)	73.77
Silt (%)	12
Clay (%)	13.5

### Organic fertilizer (FR) Preparation

Analyzed sludge/sediment was collected after fish harvesting from a Nile tilapia (*Oreochromis niloticus*) earthen pond (≈ 2000 m²) at a fish farm owned by Mariut Fish Farm Company, under the Authority of National Service Projects, Egyptian Armed Forces. The site is characterized by clay-alluvial soil. Compost, prepared from agricultural by-products (e.g., *B. vulgaris* leaves), was purchased from local farms in Kom El-Burka, Beheira Governorate. *B. vulgaris* as an agricultural by-product contains key bioactive substances including betalains (betanin, isobetanin, neobetanin, vulgaxanthins and indicaxanthins), flavonoids (rutin, rhamnetin, rhamnecitrin, kaempferol, and astragalin), phenolic acids (gallic, p-hydroxybenzoic, ferulic, caffeic and syringic), carotenoids (B-carotene, lycopene and lutein), minerals (Ca, Fe, Mg, Mn, K, P, Zn, Na, Cu, and Se) and vitamins (A, B1, B2, B3 B6, B9, B12, C, and E) [32, 33]. Organic fertilizers were produced following the composting procedures of Hien [34] and Thanh and Ty [35]. Wet sludge was sun-dried for seven days, then stored for use as organic fertilizer (Table 2). A mixture of 500 kg dried sludge and 500 kg compost was incubated in 10 plastic bags for 95 days at 60 °C, with mixing every 10 days.

**Table 2:** Chemical analysis of the organic fertilizer composed of fish sludge and *Beta vulgaris* leaves (FS + BVL) used in the experimental study

Analyses	(%)
Organic matter (%)	40.77
Organic carbon (%)	23.65
pH	7.73
Total nitrogen (TN)	5.21
Total phosphorus (TP)	1.55
Total potassium (TK)	0.75

### Pond Preparation

Before installing hapas, eight experimental earthen ponds (150 m² each) were completely drained and left exposed to direct sunlight for twenty days to allow complete drying and cracking of the soil. Subsequently, agricultural lime was then applied at a rate of 100 g/m². To minimize the risk of predators, pond dykes and the surrounding vegetation areas were cleared. Organic fertilizer was evenly distributed across the pond bottoms, after which water was added to a depth of 30 cm and maintained for 5–7 days until it developed a brownish coloration. One week later, hapas in treatment (4 hapa/pond) were installed, and the water level was gradually raised while monitoring water quality and observing color changes toward a lighter shade. Fish were stocked into the hapas following assessment of turbidity and microbial activity after fertilization. Each pond was equipped with two paddle-wheel aerators (3 hp each). Organic fertilizer was subsequently applied at a weekly rate of 500 kg/feddan, following the recommendations of El-Tawil [36].

### Experimental fish and rearing conditions

A total of 2,000 healthy Florida red tilapia (*Oreochromis* sp.) was used, consisting of 1,500 females (203.0 ± 1.0 g) and 500 males (277 ± 3.37 g). The broodstock was sourced from outdoor concrete tanks with a native salinity of 36‰ at the Marine Fish Rearing Unit (Kilo 21 Hatchery), Alexandria, Egypt. Fish were transferred to the experimental site and distributed into two earthen ponds (300 m³ each) for a 28-day acclimation period. During acclimation, water conditions were maintained at 27 ± 2 °C, pH 7.81, and dissolved oxygen 6.77 mg/L. Broodstock were gradually adapted to the experimental salinities (18‰ and 36‰) over four weeks by reducing salinity at a rate of 4‰ per day [37]. Fish were fed manually twice daily at 10:00 and 15:00 to apparent satiation with a commercial tilapia diet containing 30% crude protein (Aller-Aqua Egypt Co.). Underground saline water (40‰) served as the marine water source, while municipal tap water, pretreated for 24 h to remove chlorine, was used as the freshwater source.

### Experimental design

The experimental design is summarized in Table 3. For each salinity, four ponds were allocated: two ponds for unfertilized treatments (FR_0_) and two ponds for fertilized treatments (FR_1_). Within each fertilization level, broodstock were fed two dietary treatments: a plant based-fishmeal-free diet (FM_0_) and a fishmeal-enriched diet (FM_1_). This arrangement resulted in 8 treatment groups in 8 ponds. Each pond (150 m^2^/1.25 m depth) contains four hapas, treatment with four replicates, each one dimensioned 3 m × 9 m × 0.9 m with water volume 24.3 m^3^. Following acclimatization to the salinity and experimental conditions, broodfish were randomly stocked at a density of 45 females and 15 males per hapa (3:1 ratio). Florida red tilapia broodfish were fed two isoenergetic diets: a fishmeal-enriched control diet (FM_1_) and a plant-based diet without fishmeal (FM_0_) (Table 4). Both diets were evaluated under two salinity conditions (18‰ and 36‰) in fertilized and unfertilized ponds. The amino acid composition of the diets is provided in Table 5. Broodstock were manually fed the assigned diets at 1% of body weight per day for 210 days, beginning the day after stocking.

**Table 3:** Experimental design

Salinity (S)	Fertilizer (FR)	Fishmeal (FM)	
		FM_0_ (-)	FM_1_ (+)
18‱	Unfertilized (FR_0_)	T_1_ (S_18_-FR_0_-FM_0_)	T_2_ (S_18_-FR_0_-FM_1_)
	Fertilized (FR_1_)	T_3_ (S_18_-FR_1_-FM_0_)	T_4_ (S_18_-FR_1_-FM_1_)
36‱	Unfertilized (FR_0_)	T_5_ (S_36_-FR_0_-FM_0_)	T_6_ (S_36_-FR_0_-FM_1_)
	Fertilized (FR_1_)	T7 (S_36_-FR_1_-FM_0_)	T8 (S_36_-FR_1_-FM_1_)


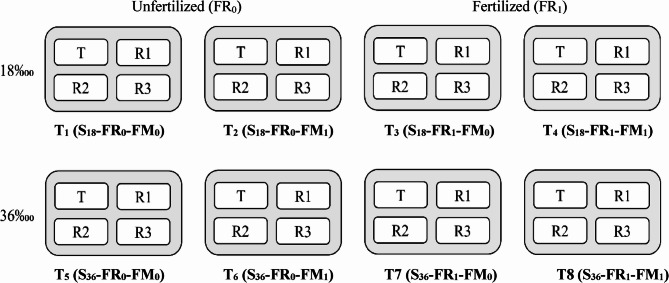


**Table 4:** Formulation and proximate composition of the tested diets (dry weight basis)

Ingredients/ Proximate analysis	Fishmeal-enriched diet (FM_1_)	Fishmeal-free diet (FM_0_)
Fishmeal (60%)	80	0
Shrimp meal (58%)	23	23
Meat and bone meal (55%)	20	20
Corn gluten meal (60%)	30	55
Soybean meal (46%)	335	345
Sesame seed meal (46%)	40	62
Wheat middlings (14%)	220	274
Rice polishing (12%)	133	152
Corn meal (8 %)	90	40
Vegetable oil	12	12
Di Calcium Phosphate	6	6
Vitamin & Mineral Premix	3	3
DI Methionine	1.25	1.25
L Lysine	1	1
Choline chloride 50%	5	5
Vitamin C (Stay C)	0.25	0.25
Antioxidant (BHT)	0.5	0.5
Total	1000	1000
Proximate analysis (%)		
Dry matter (DM)	91.06	90.92
Crude protein (CP)	31.68	31.44
Ether extract (EE)	6.51	6.37
Crude fiber (CF)	6.81	7.32
Ash	8.31	7.53
NFE^1^	46.69	47.34
Gross energy (MJ/100g)^2^	18.03	18.03

^1^NFE is nitrogen-free extract, NFE, % = 100 – (CF % + CP % + EE% + ash%);^2^Calorific value, or gross energy value of the food or substance. Using this technique the mean gross energy value for carbohydrate, lipid and protein has been estimated to be 4.1 kcal/g (17.2 kJ/g), 9.5 kcal/g (39.8 kJ/g), and 5.6 kcal/g (23.4 kJ/g) respectively [38]

**Table 5:** Amino acid composition in the experimental diets (g/kg in dry matter)

Amino acids	Fishmeal-enriched diet (FM_1_)	Fishmeal-free diet (FM_0_)
Essential amino acids		
Arginine	25.99	27.4
Histidine	11.55	11.61
Isoleucine	21.61	21.58
Leucine	39.89	38.99
Lysine	37.11	36.95
Methionine	13.8	12.93
Phenylalanine	20.77	21.11
Threonine	19.8	18.11
Valine	26.91	23.99
Non-essential amino acids		
Aspartic acid	39.88	44.89
Glutamic acid	68.99	80.12
Proline	25.11	22.99
Glycine	26.22	19.33
Alanine	24.11	21.33
Serine	22.99	3.33

### Studied traits

#### Soil, and organic fertilizer

Soil samples were collected along two diagonals of each pond. After air-drying, the samples were homogenized, and three subsamples (1 kg each) were placed in labeled plastic bags for chemical analyses. The analyses of both soil and organic fertilizers were performed at the Agriculture Directorate Laboratory, Beheira Governorate.

Soil pH was determined in a 1:5 soil-to-water suspension [38]; Total and available nitrogen were measured using the Kjeldahl method, while total and available phosphorus were quantified by spectrophotometry (ISO 11261:1995, 1995). Total potassium was analyzed through hydrofluoric acid digestion [39], and soil organic carbon was determined using the Walkley–Black wet oxidation method [40]. Unless otherwise specified, all analytical methods followed the procedures described by Houba et al. [41]

#### Water quality analyses

Water quality parameters were monitored throughout the experimental period. Salinity (‱), pH, temperature (°C), and dissolved oxygen (DO, mg/L) were measured daily in situ using a portable multimeter (Lovibond SensoDirect 150, Germany). Biweekly, 5 L water samples were collected at a depth of 0.4 m below the surface for analysis of total ammonia nitrogen (TAN), nitrate (NO₃⁻), and nitrite (NO₂⁻), following APAH [42], procedures. These measurements were performed using a Hanna Ammonia Medium Range Portable Photometer (HI96715). All measured values remained within the optimal ranges for tilapia growth [43]. Chlorophyll-a concentrations were determined according to Nurdin et al. [44], while zooplankton abundance was assessed following the method of Ndour et al. [45].

#### Reproductive performance

Reproductive performance was assessed by gently capturing brooding females with a hand net and carefully stripping the eggs from their mouths. The collected eggs were incubated separately, then counted and weighed to determine the average egg weight. The remaining eggs were frozen for subsequent chemical analysis. For each hapa, the total number of spawnings and the total number of eggs per spawning were recorded.

Reproductive performance was quantified using the following indices:

Time to first spawning (days): interval between initial stocking and the first spawning event.

Inter-spawning interval (ISI, days): time elapsed between successive spawnings to the next for females that spawned more than once [46].

Average number of spawnings per female: total number of spawnings/total number of females.

Relative fecundity (average number of eggs/spawning): total number of eggs per tank/number of spawnings.

Absolute fecundity (eggs/female): total number of eggs per tank/total number of females.

#### Egg hatchability

Eggs from the final spawning were gently removed from the females’ mouths and placed in hatching jars filled with pond water of the same salinity treatment. Hatchability percentage, incubation duration until hatching, and the time required for yolk-sac absorption were recorded. Upon completion yolk-sac absorption, 10 swim-up fries were randomly sampled, and their lengths (mm) were measured.

#### Proximate analysis

Proximate composition of the experimental diets, broodstock, and larvae was analyzed following AOAC [47] standard methods. Crude protein was determined by the Kjeldahl method after acid digestion (*N* × 6.25). Crude lipid was measured by extraction with petroleum ether using a Soxhlet apparatus. Ash content was obtained by combustion at 600 °C for 6 h in a muffle furnace. Dry matter and moisture were determined by oven-drying samples at 105 °C to constant weight. Crude fiber was analyzed according to AOAC procedures [48]. Nitrogen-free extract (NFE) or soluble carbohydrates was calculated by subtracting the sum of protein, lipid, ash, fiber, and moisture from 100. Gross energy was estimated using conversion factors of 39.54, 23.67, and 17.57 kJ/g for lipid, protein, and carbohydrate, respectively [38]. Amino acid composition of diets was analyzed by high-performance liquid chromatography (HPLC) as described by Llames and Fontaine [49].

#### Growth assessment and somatic indexes

At the end of the experiment, each fish was individually weighed and measured for total length according to sex to evaluate growth performance. The condition factor (K) was calculated following Ricker [50], based on the recorded weight and total body length. Additionally, four fish per hapa (*n* = 16 per treatment pond) were dissected to assess the hepatosomatic index (HSI), viscerosomatic index (VSI), testosomatic index (TSI), and gonadosomatic index (GSI). Growth performance, feed utilization, and the calculated indices (HSI, TSI, VSI, GSI) were determined using the following standard equations:

WG, g/fish = FBW – IBW.

ADG (g/day) = WG (g)/t (days)

$$\:\text{SR,\:\%\:=}\frac{{N}_{f}}{{N}_{i}}\times\:100$$

$$\:\text{K\:factor\:=}\frac{\text{BW}}{{L}^{3}}\times\:100$$

$$\:\text{FCR\:=}\frac{\text{FI,\:g}}{\text{WG,\:g}}$$

$$\:\text{HSI\:=}\frac{\text{Liver\:weight,\:g}}{\text{Body\:weight,\:g}}$$

$$\:\text{VSI\:=}\frac{\text{Viscera\:weight,\:g}}{\text{Body\:weight,\:g}}$$

$$\:\text{GSI\:=}\frac{\text{Gonad\:weight,\:g}}{\text{Body\:weight,\:g}}$$

$$\:\text{TSI\:=}\frac{\text{Tests\:weight,\:g}}{\text{Body\:weight,\:g}}$$

Where: WG.: weight gain; FBW.: final body weight; IBW.: initial body weight; ADG.: average daily gain; t: trial period; SR.: survival rate; N_f_: final number; N_i_: initial number; K factor: condition factor; BW: Body weight; L: fish length; FCR.: feed conversion ratio; HSI: Hepatosomatic Index; VSI: Viscerosomatic Index; TSI: testosomatic Index; GSI: Gonadosomatic Index.

#### Blood biochemical evaluation

Following administration of the experimental diets, all fish in the hapa were subjected to a 24-hour fasting period prior to blood sampling. From each experimental group, 3 females and 3 males per hapa (*n* = 12♀ and 12♂ per treatment) were sampled for serum collection. Fish were anesthetized with 50 mg/L clove oil [51], after which blood was drawn from the caudal vein using sterile syringes. The collected blood was transferred into tubes without anticoagulants for subsequent biochemical analysis.

Serum testosterone and progesterone concentrations were determined using commercial ELISA test kits, following the procedure described by Tietz [52]. The technique outlined by Whitehead et al. [53] was employed to quantify serum urea and uric acid levels. Aspartate aminotransferase (AST) and alanine aminotransferase (ALT) were analyzed according to the methodology established by Bergmeyer et al. [54]. The total cholesterol concentration was measured via free cholesterol and cholesteryl ester enzyme tests [55]. Serum stress indicators cortisol and creatinine were estimated following the methodology of Sadoul and Geffroy [56] and Heinegård and Tiderström [57], respectively.

#### Antioxidant activity assessment

Antioxidant biomarkers were evaluated using diagnostic kits acquired from Biodiagnostic Co., Cairo, Egypt. The antioxidant enzyme assays, including catalase (CAT) [58], superoxide dismutase (SOD) [59], and glutathione peroxidase (GP_X_) [60], were assessed via colorimetric techniques. The concentration of malondialdehyde (MDA) in liver samples was quantified utilizing a thiobarbituric acid assay [61]. In brief, CAT activity was determined from the rate of hydrogen peroxide decomposition, MDA levels were quantified via reaction with thiobarbituric acid at 95 °C, and GPx activity was analyzed after centrifugation for 30 min at 4 °C.

#### Serum immunological properties assessment

Serum total protein (TP; g/dL) was determined using the biuret method outlined by Doumas et al. [62]. The albumin concentration (g dL⁻¹) was assessed using the bromocresol green method as outlined by Reinhold [63], whereas the globulin concentration (g dL⁻¹) was derived by subtracting the albumin concentration from the total protein concentration. Serum lysozyme activity (U mg⁻¹) was measured according to the methodology outlined by Kim and Austin [64].

#### Digestive enzymes evaluation

The activities of amylase, protease and lipase were evaluated following the protocols established by Zamani et al. [65].

### Statistical analysis

Data were presented as mean ± standard error of the mean (SEM). Statistical analyses were performed using SPSS software (version 26). A one-way ANOVA followed by Tukey’s post hoc test was applied to identify differences among treatments. In addition, a three-way ANOVA was conducted to evaluate the effects of fertilizer (FR), salinity (S), and diet type (FM_0_ and FM_1_), as well as their interactions, on all measured parameters. Normality of data distribution was verified using the Shapiro–Wilk test. Differences were considered statistically significant at *P* < 0.05.

## Results

### Soil and water analyses of the red tilapia experimental ponds

Table 6 summarizes the soil and water characteristics of red tilapia ponds under different fertilization, salinity, and dietary treatments. The one-way ANOVA showed significant differences (*P* ≤ 0.05) among all parameters, while the three-way ANOVA (Table 7) indicated that fertilizer, salinity, and diet each had significant independent effects. Interaction effects were mostly non-significant (*P* > 0.05), except for soil organic carbon. Overall, fertilizer and fishmeal inclusion had stronger impacts than salinity.

**Table 6:** Results of soil and water analyses in red tilapia experimental ponds tested with different levels of FS + BVL fertilizer (FR), salinity (S), and fishmeal-enriched (FM_1_)/fishmeal-free (FM_0_) diets

Evaluated parameters	Treatments^1^								
	FR_0_S_18_FM_0_	FR_0_S_18_FM_1_	FR_0_S_36_FM_0_	FR_0_S_36_FM_1_	FR_1_S_18_FM_0_	FR_1_S_18_FM_1_	FR_1_S_36_FM_0_	FR_1_S_36_FM_1_	*P*-value
Soil parameters									
Organic matter (%)	2.388^de^	3.163^c^	2.255^e^	2.973^cd^	4.365^b^	5.833^a^	4.518^b^	5.788^a^	0.001
Total carbon (%)	5.558^d^	6.473^cd^	6.610^c^	7.133^c^	8.738^b^	9.388^ab^	9.925^a^	9.738^ab^	0.001
Organic carbon (%)	0.995^c^	1.088^c^	0.893^c^	2.185^b^	3.488^a^	3.615^a^	3.383^a^	3.453^a^	0.001
Total nitrogen (mg/100 g)	134.50^cd^	145.25^c^	131.75^d^	141.75^cd^	159.25^b^	175.75^a^	156.50^b^	174.75^a^	0.001
Available nitrogen (mg/100 g)	10.72^c^	11.53^c^	11.58^c^	11.82^c^	13.26^b^	16.49^a^	13.11^b^	16.74^a^	0.001
Total phosphorus (mg/100 g)	17.75^d^	20.25^cd^	19.50^cd^	21.50^c^	32.75^b^	34.50^ab^	34.25^ab^	36.50^a^	0.001
Available phosphorus (mg/100 g)	0.600^b^	0.680^b^	0.713^b^	0.815^b^	1.228^a^	1.455^a^	1.448^a^	1.453^a^	0.001
PH soil	7.37^bcd^	7.48^abc^	7.80^a^	7.61^ab^	7.60^ab^	7.12^d^	7.54^abc^	7.25^cd^	0.001
Water parameters									
DO (mg/L)	5.64^e^	5.30^f^	5.76^de^	5.47^ef^	6.03^cd^	6.13^bc^	6.59^a^	6.35^ab^	0.001
PH	7.50^cde^	7.39^e^	7.75^bc^	7.40^de^	7.76^bc^	7.74^bcd^	8.12^a^	8.04^ab^	0.001
TAN (mg/L)	0.798^c^	0.970^b^	1.020^ab^	1.133^a^	0.608^d^	0.710^cd^	0.708^cd^	0.755^cd^	0.001
NO_2_ (mg/L)	0.036^c^	0.045^b^	0.046^b^	0.053^a^	0.029^d^	0.030^cd^	0.028^d^	0.027^d^	0.001
NO_3_ (mg/L))	0.319^cd^	0.223^e^	0.226^e^	0.200^e^	0.430^a^	0.364^b^	0.361^bc^	0.309^d^	0.001
Chlorophyll a (mg m-^3^)	1780.3^cd^	1500.8^d^	733.3^e^	842.5^e^	2507.3^b^	3161.8^a^	2324.5^bc^	1628.5^d^	0.001
Zooplankton (# of individuals)	884.3^e^	1124.8^cde^	573.8^f^	996.8^de^	1313.8^bc^	1595.5^a^	1161.8^bcd^	1384.8^ab^	0.001

^1^ Treatments: FR_0_S_18_FM_0_ = ponds with no fertilizer (FR) at salinity (S) 18ppt and fish fed diet without fishmeal (FM); FR_0_S_18_FM_1_ = nonfertilized ponds at salinity 18ppt and fish fed diet with FM; FR_0_S_36_FM_0_ = nonfertilized ponds at salinity 36ppt and fish fed diet without FM; FR_0_S_36_FM_1_ = nonfertilized ponds at salinity 36ppt and fish fed diet with FM; FR_1_S_18_FM_0_ = fertilized ponds at salinity 18ppt and fish fed diet without FM; FR_1_S_18_FM_1_ = fertilized ponds at salinity 18ppt and fish fed diet with FM; FR_1_S_36_FM_0_ = fertilized ponds at salinity 36ppt and fish fed diet without FM, and FR_1_S_36_FM_1_ = fertilized ponds at salinity 36ppt and fish fed diet with FM. DO = Dissolved oxygen; TAN = total ammonia nitrogen: No_2_ = nitrite; No_3_ = nitrate; FS + BVL = fish sludge + *Beta vulgaris* leaves

**Table 7:** Results of three-way ANOVA analysis with SPSS for the soil and water analyses in red tilapia experimental ponds tested with different levels of FS + BVL fertilizer (FR), salinity (S), and fishmeal-enriched (FM_1_)/fishmeal-free (FM_0_) diets

Dependent Variable	*P*-value of the main effects			*P*-value of the interaction effects			
	fertilizer effect	Salinity effect	Fishmeal effect	FR*S	FR*FM	S*FM	FR*S*FM
Soil analyses							
Organic matter (%)	0.000	0.614	0.000	0.317	0.007	0.550	0.742
Total carbon (%)	0.000	0.000	0.006	0.785	0.138	0.064	0.490
Organic carbon (%)	0.000	0.005	0.000	0.000	0.000	0.000	0.000
Total nitrogen (mg/100 g)	0.000	0.146	0.000	0.711	0.046	0.882	0.711
Available nitrogen (mg/100 g)	0.000	0.071	0.000	0.124	0.000	0.803	0.159
Total phosphorus (mg/100 g)	0.000	0.001	0.000	0.784	0.784	1.000	0.584
Available phosphorus (mg/100 g)	0.000	0.006	0.012	0.846	0.747	0.204	0.122
PH soil	0.001	0.005	0.000	0.027	0.002	0.611	0.024
Water analyses							
DO (mg/L)	0.000	0.000	0.001	0.019	0.016	0.143	0.053
PH	0.000	0.000	0.014	0.078	0.094	0.170	0.407
TAN (mg/L)	0.000	0.000	0.000	0.015	0.152	0.220	0.957
NO_2_ (mg/L)	0.000	0.000	0.000	0.000	0.000	0.251	1.000
NO_3_ (mg/L))	0.000	0.000	0.000	0.758	0.923	0.003	0.039
Chlorophyll a (mg m-^3^)	0.000	0.000	0.001	0.976	0.722	0.013	0.800
Zooplankton (# of individuals)	0.000	0.000	0.000	0.617	0.303	0.420	0.121

FR*S= interaction of fertilizer and salinity; FR*FM= interaction of fertilizer and fishmeal; S*FM= interaction of salinity and fishmeal; FR*S*FM= interaction of fertilizer, salinity and fishmeal; DO= Dissolved oxygen; TAN= total ammonia nitrogen: No_2_= nitrite; No_3_ = nitrate; FS+BVL= fish sludge + *Beta vulgaris* leaves

### Growth, feed utilization, body composition, and somatic indexes of red tilapia

Table 8 presents the growth, feed utilization, body composition, and somatic indices of red tilapia, while Table 9 shows the three-way ANOVA results. No significant differences (*P* > 0.05) were found in growth or feed efficiency, but somatic indices and body composition varied significantly (*P* ≤ 0.05) among treatments. Salinity mainly affected growth, whereas fertilizer and diet had stronger effects on body composition. The most notable interaction was FR*FM_1_, followed by FR and S*FM_1_, with the three-way interaction being least significant. FS + BVL treatments improved GSI (♀), TSI (♂), and crude protein (♀) compared to controls, except for the FR_0_S_18_FM_1_ group.

**Table 8:** Results of growth performance, feed utilization, body chemical composition and somatic indexes of red tilapia broodstock (♀ and ♂) tested with different levels of FS+BVL fertilizer (FR), salinity (S), and fishmeal-enriched (FM_1_)/fishmeal-free (FM_0_) diets

Evaluated parameters									Treatments^1^	*P*-value
		FR_0_S_18_FM_0_	FR_0_S_18_FM_1_	FR_0_S_36_FM_0_	FR_0_S_36_FM_1_	FR_1_S_18_FM_0_	FR_1_S_18_FM_1_	FR_1_S_36_FM_0_	FR_1_S_36_FM_1_	
**Growth performance & feed utilization** **(**♀)										
IBW, g	♀	200.5	203.25	201.75	203.5	202.25	201.5	202.5	201.5	0.218
FBW, g	♀	262.45	274.65	274.2	272.28	262.1	268.18	272.85	270.28	0.219
WG, g	♀	61.95	71.4	72.45	68.78	59.85	66.68	70.35	68.78	0.056
ADG	♀	0.295	0.34	0.345	0.328	0.285	0.318	0.335	0.328	0.056
FCR	♀	1.448	0.98	1.315	1.243	1.225	1.205	1.215	1.093	0.242
K	♀	2.39^cd^	2.79^a^	2.03^e^	2.26^d^	2.69^ab^	2.79^a^	2.49^bc^	2.55^bc^	0.001
**Body composition** (♀)										
Moisture	♀	71.87^a^	71.88^a^	70.71^b^	71.83^a^	71.84^a^	71.85^a^	71.84^a^	71.84^a^	0.008
Dry matter	♀	28.14^b^	28.13^b^	29.30^a^	28.18^b^	28.16^b^	28.15^b^	28.16^b^	28.17^b^	0.008
Crude protein	♀	58.84^c^	60.43^a^	59.39^b^	59.79^ab^	60.66^a^	60.59^a^	60.27^ab^	60.75^a^	0.001
Crude Lipid	♀	21.04^a^	20.50^ab^	21.06^a^	20.57^ab^	20.20^b^	20.29^b^	20.09^b^	20.33^b^	0.001
Ash	♀	15.96^c^	17.76^ab^	16.46^bc^	18.48^a^	18.30^a^	18.12 ^a^	17.22^abc^	17.84^a^	0.001
**Somatic and gonad indexes** **(** ♀&♂)										
HSI	♀	2.4125^b^	2.3075^b^	2.7675^a^	2.6050^a^	2.3425^b^	2.1975^b^	2.2750^b^	2.3500^b^	0.001
HSI	♂	1.6425^b^	1.5100^c^	1.7575^a^	1.6300^b^	1.5175^c^	1.4800^c^	1.5075^c^	1.5350^c^	0.001
VSI	♀	7.2750^b^	6.6350^cd^	8.0000^a^	7.4575^b^	6.8125^c^	6.4600^d^	6.5400^cd^	6.6200^cd^	0.001
VSI	♂	6.3375^b^	5.6525^de^	6.9200^a^	6.4250^b^	6.0275^c^	5.5650^e^	5.7300^cde^	5.8975^cd^	0.001
GSI	♀	5.2900^b^	5.9025^a^	4.3075^c^	5.3950^b^	5.9350^a^	5.9300^a^	5.9000^a^	5.8725^a^	0.001
TSI	♂	1.4675^b^	2.0875^a^	0.9850^c^	1.6425^b^	1.9625^a^	2.1500^a^	2.1100^a^	2.0725^a^	0.001

^1^ Treatments: FR_0_S_18_FM_0_= ponds with no fertilizer (FR) at salinity (S) 18ppt and fish fed diet without fishmeal (FM); FR_0_S_18_FM_1_= nonfertilized ponds at salinity 18ppt and fish fed diet with FM; FR_0_S_36_FM_0_= nonfertilized ponds at salinity 36ppt and fish fed diet without FM; FR_0_S_36_FM_1_ = nonfertilized ponds at salinity 36ppt and fish fed diet with FM; FR_1_S_18_FM_0_ = fertilized ponds at salinity 18ppt and fish fed diet without FM; FR_1_S_18_FM_1_ = fertilized ponds at salinity 18ppt and fish fed diet with FM; FR_1_S_36_FM_0_ = fertilized ponds at salinity 36ppt and fish fed diet without FM, and FR_1_S_36_FM_1_ = fertilized ponds at salinity 36ppt and fish fed diet with FM. IBW= initial body weight; FBW= final body weight; WG= weight gain; ADG= average daily gain; t: trial period; SGR%= specific growth rate; SR= survival rate; K = condition factor; FCR. = feed conversion ratio; HSI= Hepatosomatic Index; VSI= Viscerosomatic Index; TSI= testosomatic Index; GSI= Gonadosomatic Index; FS+BVL= fish sludge +*Beta vulgaris*leaves.

**Table 9:** Results of three-way ANOVA analysis with SPSS for growth performance, feed utilization, body chemical composition and somatic indexes of red tilapia broodstock (♀ and ♂) tested with different levels of FS + BVL fertilizer (FR), salinity (S), and fishmeal-enriched (FM_1_)/fishmeal-free (FM_0_) diets

Dependent Variable		*P*-value of the main effects			*P*-value of the interaction effects			
		fertilizer effect	Salinity effect	Fishmeal effect	FR*S	FR*FM	S*FM	FR*S*FM
Growth performance & feed utilization( ♀)								
I.BW, g		0.591	0.453	0.243	0.591	0.012	0.591	0.747
FBW, g		0.221	0.011	0.102	0.671	0.411	0.010	0.505
WG, g		0.291	0.021	0.195	0.573	0.950	0.016	0.573
ADG		0.291	0.021	0.195	0.573	0.950	0.016	0.573
FCR		0.460	0.982	0.049	0.451	0.240	0.384	0.144
K factor		0.000	0.000	0.000	0.001	0.001	0.129	0.287
Body composition ( ♀)								
Moisture		0.080	0.051	0.069	0.056	0.070	0.081	0.073
Dry matter		0.078	0.053	0.071	0.058	0.070	0.081	0.072
Crude protein		0.007	0.628	0.000	0.004	0.001	0.623	0.238
Crude lipid		0.010	0.957	0.002	0.101	0.007	0.114	0.856
Crude ash		0.002	0.867	0.000	0.004	0.000	0.225	0.466
Somatic indexes ( ♀ & ♂)								
HSI	♀	0.000	0.000	0.023	0.000	0.168	0.253	0.057
HSI	♂	0.000	0.000	0.000	0.000	0.000	0.126	0.187
VSI	♀	0.000	0.000	0.000	0.000	0.000	0.017	0.116
VSI	♂	0.000	0.000	0.000	0.000	0.000	0.001	0.047
TSI	♂	0.000	0.000	0.000	0.000	0.000	0.218	0.089
GSI	♀	0.000	0.000	0.000	0.000	0.000	0.009	0.004

FR*S= interaction of fertilizer and salinity; FR*FM= interaction of fertilizer and fishmeal; S*FM= interaction of salinity and fishmeal; FR*S*FM= interaction of fertilizer, salinity and fishmeal; IBW= initial body weight; FBW= final body weight; WG= weight gain; ADG= average daily gain; t: trial period; SGR= specific growth rate; SR= survival rate; K factor= condition factor; FCR. = feed conversion ratio; HSI= Hepatosomatic Index; V.S. I= Viscerosomatic Index; T.S.I.= testosomatic Index; G.S.I.= Gonadosomatic Index; FS+BVL= fish sludge + *Beta vulgaris* leaves

### Serum cholesterol, liver enzymes, kidney health indicators, and cortisol activity in red tilapia broodstock (♀ and ♂).

Table 10 shows significant differences (*P* ≤ 0.05) in cortisol, liver metabolism, and kidney function indices among treatments, while Table 11 details the main and interactive effects. Fertilizer had the strongest influence, followed by diet (FM_1_), whereas salinity had the least effect. The FR*FM_1_ interaction was most significant, exceeding FR*S and S*FM_1_, with the three-way interaction generally non-significant except for male T-cholesterol, liver enzymes (ALT, AST), and creatinine. The FR_1_S_18_FM_1_ treatment recorded the lowest liver and kidney stress levels, closely followed by FR_1_S_18_FM_0_, with no significant differences between them.

**Table 10:** Results of serum cholesterol, liver and kidney metabolic activity and cortisol levels in red tilapia broodstock (♀ and ♂) tested with different levels of FS + BVL fertilizer (FR), salinity (S), and fishmeal-enriched (FM_1_)/fishmeal-free (FM_0_) diets

Evaluated parameters			Treatments^1^								*P*-value
			FR_0_S_18_FM_0_	FR_0_S_18_FM_1_	FR_0_S_36_FM_0_	FR_0_S_36_FM_1_	FR_1_S_18_FM_0_	FR_1_S_18_FM_1_	FR_1_S_36_FM_0_	FR_1_S_36_FM_1_	
Cholesterol (mg/dl)		♀	246.25^ab^	234.50^b^	253.75^a^	233.75^b^	118.25^c^	119.75^c^	129.25^c^	126.50^c^	0.001
		♂	239.50^ab^	232.75^b^	248.50^a^	227.75^b^	116.25^cd^	107.75^d^	124.50^c^	121.75^cd^	0.001
Liver enzymes and cortisol activity											
ALT (IU/L)	♀		31.958^d^	31.790^d^	46.278^b^	59.390^a^	31.125^d^	29.958^d^	38.975^c^	31.678^d^	0.001
	♂		32.603^d^	32.435^d^	45.043^b^	56.570^a^	31.125^d^	30.583^d^	38.478^c^	31.915^d^	0.001
AST (IU/L)	♀		60.360^de^	59.360^e^	75.000^b^	88.253^a^	56.750^f^	54.543^g^	68.008^c^	61.015^d^	0.001
	♂		62.33^d^	61.16^d^	75.90^b^	88.44^a^	58.21^e^	54.63^f^	68.52^c^	61.13^d^	0.001
cortisol (pg mL^−1^)	♀		224.13^ab^	218.56^b^	242.41^a^	219.81^b^	175.67^c^	173.21^c^	186.83^c^	172.98^c^	0.001
	♂		216.96^ab^	210.00^ab^	236.89^a^	210.59^ab^	198.50^b^	193.44^b^	181.75^b^	194.22^b^	0.003
Kidney function indicators											
Urea (mg/dl)	♀		40.50^a^	40.50^a^	42.60^a^	39.61^a^	34.08^b^	34.03^b^	35.04^b^	34.14^b^	0.001
	♂		41.11^a^	39.22^b^	42.23^a^	39.74^b^	36.40^c^	36.31^c^	37.32^c^	36.36^c^	0.001
Uric Acid (mg/dl)	♀		2.61^a^	2.58^a^	3.07^a^	2.59^a^	1.71^b^	1.68^b^	1.89^b^	1.70^b^	0.001
	♂		2.845^ab^	2.683^b^	3.085^a^	2.675^b^	2.140^c^	2.045^c^	2.243^c^	2.100^c^	0.001
Creatinine (mg/dl)	♀		1.050^c^	0.915^d^	1.313^a^	1.210^ab^	1.243^ab^	0.773^e^	1.138^bc^	1.023^cd^	0.001
	♂		1.120^c^	0.985^d^	1.383^a^	1.280^ab^	1.313^ab^	0.843^e^	1.208^bc^	1.093^cd^	0.001

^1^Treatments: FR_0_S_18_FM_0_= ponds with no fertilizer (FR) at salinity (S) 18ppt and fish fed diet without fishmeal (FM); FR_0_S_18_FM_1_= nonfertilized ponds at salinity 18ppt and fish fed diet with FM; FR_0_S_36_FM_0_= nonfertilized ponds at salinity 36ppt and fish fed diet without FM; FR_0_S_36_FM_1_= nonfertilized ponds at salinity 36ppt and fish fed diet with FM; FR_1_S_18_FM_0_= fertilized ponds at salinity 18ppt and fish fed diet without FM; FR_1_S_18_FM_1_= fertilized ponds at salinity 18ppt and fish fed diet with FM; FR_1_S_36_FM_0_= fertilized ponds at salinity 36ppt and fish fed diet without FM, and FR_1_S_36_FM_1_= fertilized ponds at salinity 36ppt and fish fed diet with FM.; ALT = glutamic-pyruvic transaminase; AST = aspartate aminotransferase; FS+BVL= fish sludge + *Beta vulgaris*leaves

**Table 11:** Results of three-way ANOVA analysis with SPSS for serum cholesterol, liver and kidney metabolic activity and cortisol levels in red tilapia broodstock (♀ and ♂) tested with different levels of FS + BVL fertilizer (FR), salinity (S), and fishmeal-enriched (FM_1_)/fishmeal-free (FM_0_) diets

Dependent Variable		*P*-value of the main effects			*P*-value of the interaction effects			
		fertilizer effect	Salinity effect	Fishmeal effect	FR*S	FR*FM	S*FM	FR*S*FM
T. cholesterol (mg/dl)	♀	0.000	0.006	0.000	0.191	0.001	0.139	0.629
	♂	0.000	0.007	0.000	0.053	0.082	0.367	0.037
ALT (IU/L)	♀	0.000	0.000	0.003	0.000	0.000	0.000	0.000
	♂	0.000	0.000	0.004	0.000	0.000	0.000	0.000
AST (IU/L)	♀	0.000	0.000	0.002	0.000	0.000	0.000	0.000
	♂	0.000	0.000	0.810	0.000	0.000	0.000	0.000
Cortisol (pg mL-1)	♀	0.000	0.026	0.002	0.508	0.364	0.036	0.664
	♂	0.000	0.845	0.271	0.125	0.089	0.938	0.121
Urea (mg/dl)	♀	0.000	0.363	0.120	0.955	0.415	0.130	0.390
	♂	0.000	0.003	0.000	0.412	0.000	0.079	0.750
Uric Acid (mg/dl)	♀	0.000	0.092	0.068	0.487	0.455	0.112	0.440
	♂	0.000	0.017	0.000	0.628	0.038	0.065	0.203
Creatinine (mg/dl)	♀	0.000	0.000	0.000	0.000	0.000	0.000	0.000
	♂	0.000	0.000	0.000	0.000	0.000	0.000	0.000

FR*S= interaction of fertilizer and salinity; FR*FM= interaction of fertilizer and fishmeal; S*FM= interaction of salinity and fishmeal; FR*S*FM= interaction of fertilizer, salinity and fishmeal; ALT = glutamic-pyruvic transaminase; AST = aspartate aminotransferase; FS+BVL= fish sludge + *Beta vulgaris* leaves.

### Serum immune, and antioxidant parameters in red tilapia broodstock (♀ and ♂)

Table 12 indicates significant effects (*P* ≤ 0.05) of fertilizer, salinity, and diet on serum immune and antioxidant parameters of broodstock, with Table 13 detailing their main and interaction effects. The highest lysozyme, TP, globulin, GPx, CAT, and SOD levels and the lowest MDA values occurred in the FR_1_S_18_FM_1_ and FR_1_S_18_FM_0_ treatments. Fertilizer showed the strongest influence, followed by salinity and diet. The combined effect of all three factors notably enhanced TP, albumin, and globulin in both sexes and significantly affected CAT♂, SOD♂, and MDA (♀ and ♂).

**Table 12:** Results of serum immune and antioxidant parameters in red tilapia broodstock (♀ and ♂) tested with different levels of FS + BVL fertilizer (FR), salinity (S), and fishmeal-enriched (FM_1_)/fishmeal-free (FM_0_) diets

Evaluated parameters		Treatments^1^								*P*-value
		FR_0_S_18_FM_0_	FR_0_S_18_FM_1_	FR_0_S_36_FM_0_	FR_0_S_36_FM_1_	FR_1_S_18_FM_0_	FR_1_S_18_FM_1_	FR_1_S_36_FM_0_	FR_1_S_36_FM_1_	
Serum immune parameters										
Lysozyme (µg/ml)	♀	0.400^bcd^	0.443^abcd^	0.328^d^	0.393^cd^	0.545^ab^	0.590^a^	0.538^abc^	0.575^a^	0.001
	♂	0.435^b^	0.393^b^	0.338^b^	0.413^b^	0.593^a^	0.603^a^	0.570^a^	0.598^a^	0.001
Total protein (g/dl)	♀	3.603^c^	3.618^bc^	3.160^e^	2.698^f^	3.693^ab^	3.763^a^	3.413^d^	3.668^bc^	0.001
	♂	3.413^d^	3.663^bc^	3.180^e^	2.810^f^	3.785^ab^	3.808^a^	3.573^c^	3.643^c^	0.001
Albumin (g/dl)	♀	0.498^cd^	0.440^d^	0.623^b^	0.763^a^	0.420^d^	0.435^d^	0.548^bc^	0.470^cd^	0.001
	♂	0.660^c^	0.558^e^	0.708^b^	0.883^a^	0.620^cd^	0.485^f^	0.600^d^	0.498^f^	0.001
Globulin (g/dl)	♀	3.108^c^	3.180^b^	2.538^e^	1.933^f^	3.273^a^	3.330^a^	2.868^d^	3.198^b^	0.001
	♂	2.915^c^	3.108^b^	2.473^d^	1.923^e^	3.165^b^	3.323^a^	2.810^c^	3.145^b^	0.001
Antioxidant parameters										
GP_x_	♀	1.510^c^	2.198^a^	1.025^d^	1.840^b^	2.195^a^	2.250^a^	2.073^a^	2.168^a^	0.001
	♂	1.558^e^	2.098^bc^	1.138^f^	1.780^d^	2.100^abc^	2.180^ab^	2.013^c^	2.243^a^	0.001
CAT	♀	14.38^c^	16.90^a^	12.37^d^	15.61^b^	16.90^a^	17.10^a^	16.54^a^	16.85^a^	0.001
	♂	14.52^e^	16.93^bc^	12.62^f^	15.31^d^	16.92^bc^	17.04^b^	16.33^c^	45.67^a^	0.001
SOD	♀	54.39^c^	65.64^a^	45.74^d^	60.73^b^	65.61^a^	66.22^a^	63.89^ab^	65.11^a^	0.001
	♂	53.85^c^	63.97^a^	45.34^d^	59.73^b^	63.90^a^	64.51^a^	62.60^a^	54.44^c^	0.001
MDA	♀	0.890^b^	0.550^c^	1.190^a^	0.640^c^	0.550^c^	0.538^c^	0.578^c^	0.550^c^	0.001
	♂	0.863^c^	0.528^e^	1.140^b^	1.860^a^	0.528^e^	0.500^e^	0.563^e^	0.660^d^	0.001

^1^Treatments: FR_0_S_18_FM_0_= ponds with no fertilizer (FR) at salinity (S) 18ppt and fish fed diet without fishmeal (FM); FR_0_S_18_FM_1_= nonfertilized ponds at salinity 18ppt and fish fed diet with FM; FR_0_S_36_FM_0_= nonfertilized ponds at salinity 36ppt and fish fed diet without FM; FR_0_S_36_FM_1_= nonfertilized ponds at salinity 36ppt and fish fed diet with FM; FR_1_S_18_FM_0_= fertilized ponds at salinity 18ppt and fish fed diet without FM; FR_1_S_18_FM_1_= fertilized ponds at salinity 18ppt and fish fed diet with FM; FR_1_S_36_FM_0_= fertilized ponds at salinity 36ppt and fish fed diet without FM, and FR_1_S_36_FM_1_= fertilized ponds at salinity 36ppt and fish fed diet with FM.; MAD = malondialdehyde; SOD = superoxide dismutase; CAT = catalase; GPx = Activity of glutathione peroxidase; FS+BVL= fish sludge +*Beta vulgaris*leaves.

**Table 13:** Results of three-way ANOVA analysis with SPSS for serum immune and antioxidant parameters in red tilapia broodstock (♀ and ♂) tested with different levels of FS + BVL fertilizer (FR), salinity (S), and fishmeal-enriched (FM_1_)/fishmeal-free (FM_0_) diets

Dependent Variable		*P*-value of the main effects			*P*-value of the interaction effects			
		fertilizer effect	Salinity effect	Fishmeal effect	FR*S	FR*FM	S*FM	FR*S*FM
Lysozyme (µg/ml)	♀	0.000	0.125	0.048	0.284	0.786	0.871	0.745
	♂	0.000	0.284	0.472	0.607	0.959	0.172	0.307
Total protein (g/dl)	♀	0.000	0.000	0.015	0.000	0.000	0.000	0.000
	♂	0.000	0.000	0.743	0.000	0.000	0.006	0.000
Albumin (g/dl)	♀	0.000	0.000	0.749	0.000	0.028	0.102	0.000
	♂	0.000	0.002	0.128	0.001	0.007	0.007	0.028
Globulin (g/dl)	♀	0.000	0.000	0.000	0.000	0.000	0.000	0.000
	♂	0.000	0.000	0.071	0.000	0.000	0.000	0.000
GPx	♀	0.000	0.000	0.000	0.000	0.000	0.153	0.448
	♂	0.000	0.000	0.000	0.000	0.000	0.007	0.587
CAT	♀	0.000	0.000	0.000	0.000	0.000	0.089	0.207
	♂	0.000	0.000	0.000	0.000	0.000	0.000	0.000
SOD	♀	0.000	0.000	0.000	0.000	0.000	0.032	0.113
	♂	0.000	0.000	0.000	0.325	0.000	0.003	0.000
MDA	♀	0.000	0.000	0.000	0.000	0.000	0.015	0.032
	♂	0.000	0.000	0.000	0.000	0.000	0.000	0.000

FR*S= interaction of fertilizer and salinity; FR*FM= interaction of fertilizer and fishmeal; S*FM= interaction of salinity and fishmeal; FR*S*FM= interaction of fertilizer, salinity and fishmeal; MAD = malondialdehyde; SOD = superoxide dismutase; CAT = catalase; GPx = Activity of glutathione peroxidase; FS+BVL= fish sludge + *Beta vulgaris* leaves.

### Serum digestive enzyme and reproductive-related hormones activity in red tilapia broodstock (♀ and ♂) 

**Fig. 1:**
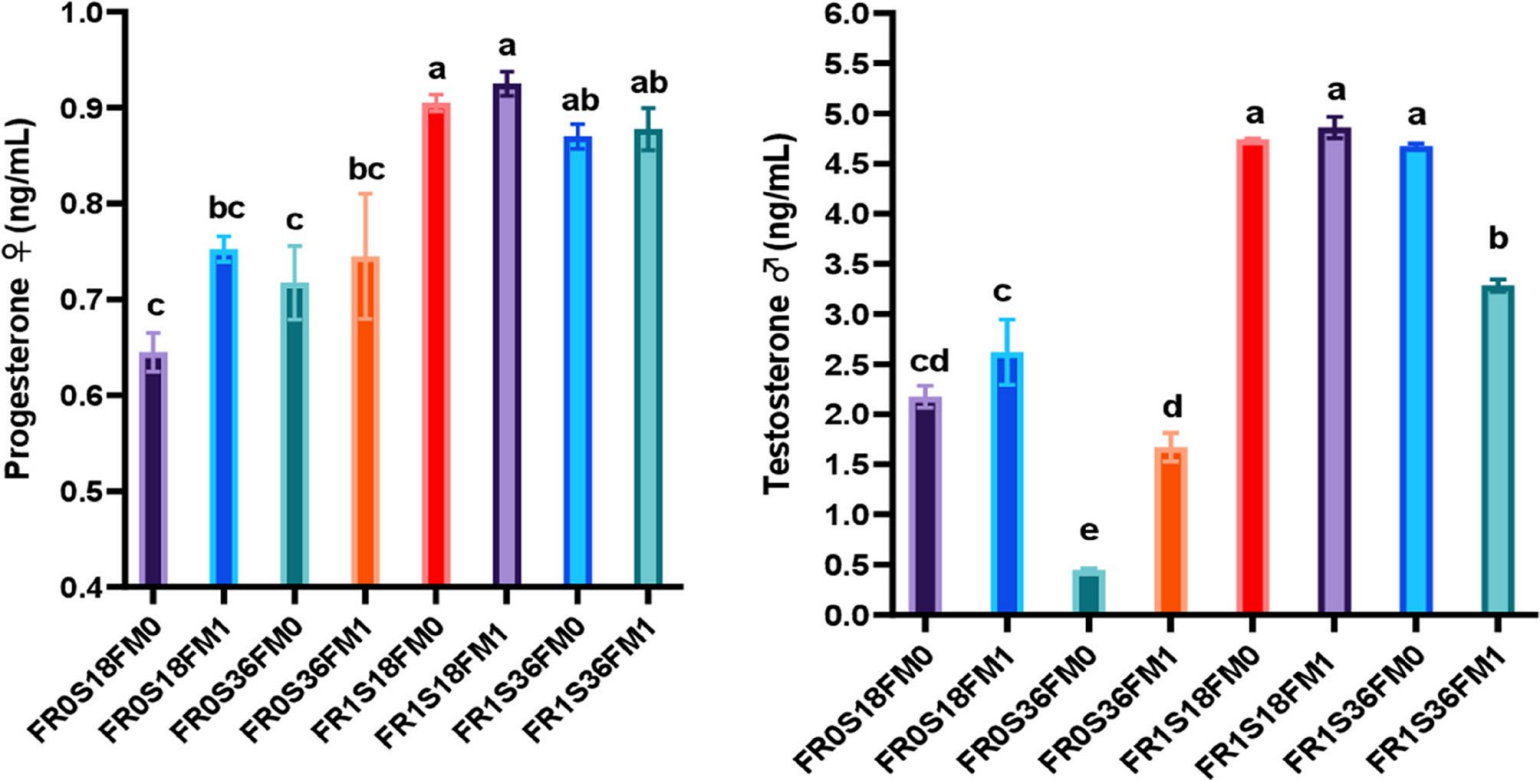
Serum reproductive hormones (progesterone ♀ and testosterone ♂) activity in red tilapia broodstock tested with different levels of FS+BVL fertilizer, salinity (18 and 36ppt), and fishmeal enriched (FM_1_)/fishmeal-free (FM_0_) diets. Where, FS+BVL= fish sludge + *Beta vulgaris* leaves

Table 14; Fig. 1 show significant effects (*P* ≤ 0.05) of fertilizer, salinity, and diet on serum reproductive hormones and digestive enzyme activity in red tilapia broodstock, with Table 15 detailing the main and interaction effects. Fertilizer had the strongest influence, followed by diet, while salinity had the least. The FR*FM_1_ interaction was the most pronounced, with FR_1_S_18_FM_1_ showing the highest digestive enzyme levels, followed by FR_1_S_18_FM_0_. All FS + BVL-treated groups exhibited higher progesterone and testosterone levels than unfertilized fish.

**Table 14:** Results of serum digestive enzymes and reproductive-related hormones in red tilapia broodstock (♀ and ♂) tested with different levels of FS + BVL fertilizer (FR), salinity (S), and fishmeal-enriched (FM_1_)/fishmeal-free (FM_0_) diets

Evaluated parameters		Treatments^1^								*P*-value
		FR_0_S_18_FM_0_	FR_0_S_18_FM_1_	FR_0_S_36_FM_0_	FR_0_S_36_FM_1_	FR_1_S_18_FM_0_	FR_1_S_18_FM_1_	FR_1_S_36_FM_0_	FR_1_S_36_FM_1_	
Digestive enzymes										
Protease	♀	59.67^c^	61.33^bc^	51.83^e^	55.33^d^	62.67^b^	65.33^a^	58.00^cd^	61.00^bc^	0.001
	♂	60.97^cd^	62.93^bc^	53.13^f^	56.93^e^	64.17^b^	67.03^a^	59.50^d^	62.70^bc^	0.001
Amylase	♀	45.27^c^	49.19^b^	38.67^e^	42.50^d^	50.38^ab^	51.87^a^	46.50^c^	49.83^b^	0.001
	♂	46.57^d^	53.57^c^	39.97^e^	51.53^c^	51.88^c^	62.93^a^	48.00^d^	56.93^b^	0.001
Lipase	♀	46.15^c^	47.84^b^	40.50^e^	42.50^d^	49.68^ab^	51.25^a^	45.50^cd^	47.83^b^	0.001
	♂	47.45^e^	49.53^d^	41.80^f^	46.00^e^	51.18^cd^	52.95^c^	56.93^b^	62.93^a^	0.001
Serum reproductive-related hormones										
Progesterone (ng/ml)	♀	0.643^c^	0.751^bc^	0.718^c^	0.745^bc^	0.903^a^	0.925^a^	0.866^ab^	0.878^ab^	0.001
Testosterone (ng/ml)	♂	2.175^cd^	2.620^c^	0.443^e^	1.673^d^	4.745^a^	4.860^a^	4.671^a^	3.285^b^	0.001

^1^Treatments: FR_0_S_18_FM_0_= ponds with no fertilizer (FR) at salinity (S) 18ppt and fish fed diet without fishmeal (FM); FR_0_S_18_FM_1_= nonfertilized ponds at salinity 18ppt and fish fed diet with FM; FR_0_S_36_FM_0_= nonfertilized ponds at salinity 36ppt and fish fed diet without FM; FR_0_S_36_FM_1_ = nonfertilized ponds at salinity 36ppt and fish fed diet with FM; FR_1_S_18_FM_0_ = fertilized ponds at salinity 18ppt and fish fed diet without FM; FR_1_S_18_FM_1_ = fertilized ponds at salinity 18ppt and fish fed diet with FM; FR_1_S_36_FM_0_ = fertilized ponds at salinity 36ppt and fish fed diet without FM, and FR_1_S_36_FM_1_= fertilized ponds at salinity 36ppt and fish fed diet with FM; FS+BVL= fish sludge +*Beta vulgaris*leaves.

**Table 15:** Results of three-way ANOVA analysis with SPSS for serum digestive enzymes and reproductive-related hormones in red tilapia broodstock (♀ and ♂) tested with different levels of FS + BVL fertilizer (FR), salinity (S), and fishmeal-enriched (FM_1_)/fishmeal-free (FM_0_) diets

Dependent Variable		*P*-value of the main effects			*P*-value of the interaction effects			
		Fertilizer effect	Salinity effect	Fishmeal effect	FR*S	FR*FM	S*FM	FR*S*FM
Digestive enzymes		0.000	0.867	0.058	0.084	0.243	0.291	0.423
Protease	♀	0.000	0.000	0.000	0.001	0.691	0.094	0.239
	♂	0.000	0.000	0.000	0.001	0.811	0.094	0.239
Amylase	♀	0.000	0.000	0.000	0.000	0.015	0.130	0.098
	♂	0.284	0.000	0.000	0.000	0.000	0.070	0.345
Lipase	♀	0.000	0.000	0.000	0.075	0.354	0.119	0.269
	♂	0.000	0.000	0.000	0.021	0.000	0.266	0.101
Sex hormones								
Progesterone (ng/ml)	♀	0.000	0.867	0.058	0.084	0.243	0.291	0.423
Testosterone (ng/ml)	♂	0.000	0.000	0.000	0.000	0.000	0.000	0.000

FR*S= interaction of fertilizer and salinity; FR*FM= interaction of fertilizer and fishmeal; S*FM= interaction of salinity and fishmeal; FR*S*FM= interaction of fertilizer, salinity and fishmeal; FS+BVL= fish sludge + *Beta vulgaris* leaves.

### Reproductive performance, hatching performance, egg weight, larval length and larval chemical composition of red tilapia 

**Fig. 2:**
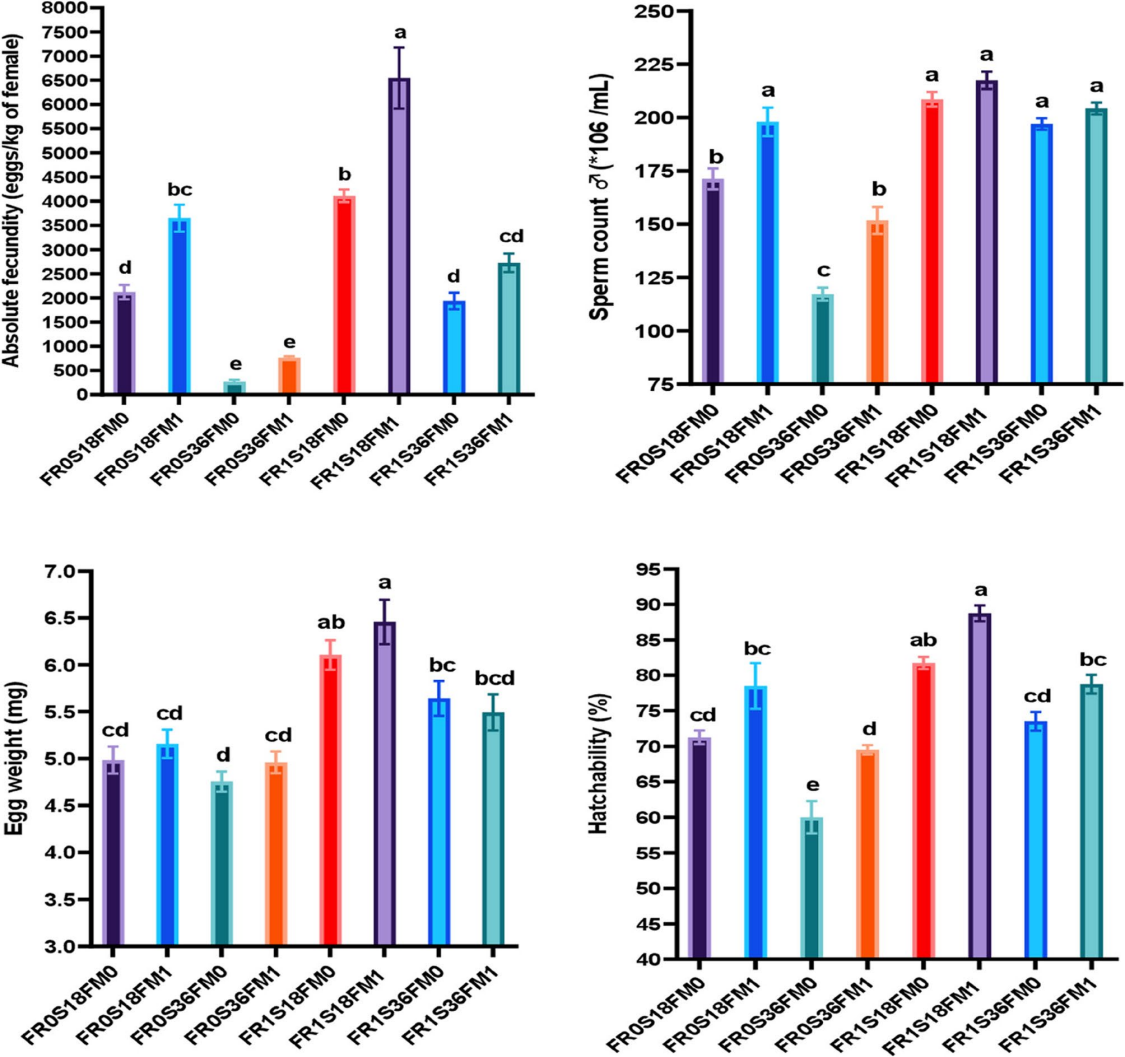
Reproductive performance, (absolute fecundity, sperm count, egg weight and hatchability) of red tilapia broodstock (♀ & ♂) tested with different levels of FS + BVL fertilizer, salinity (18 and 36ppt), and fishmeal enriched (FM_1_)/fishmeal-free (FM_0_) diets. Where, FS + BVL = fish sludge + *Beta vulgaris* leaves

Table 16; Fig. 2 show that fertilizer, salinity, and diet significantly affected most reproductive and larval performance traits of red tilapia, except egg diameter. Table 17 confirms that fertilizer had the strongest effect, followed by salinity and then diet. The FR_1_S_18_FM_1_ treatment achieved the best reproductive outcomes, including higher fecundity and spawning frequency, followed by the FR_1_S_18_FM_0_ group. Interaction effects were mostly non-significant, except for time to first spawn. Notably, the S*FM_1_ and FR*FM_1_ interactions significantly improved key spawning and larval traits such as fecundity, sperm count, yolk-sac absorption, and larval length.

**Table 16:** Results of reproductive fertility, hatchability, egg weight, larval length and larval chemical composition of red tilapia broodstock (♀ and ♂) tested with different levels of FS + BVL fertilizer (FR), salinity (S), and fishmeal-enriched (FM_1_)/fishmeal-free (FM_0_) diets

Evaluated parameters	Treatments^1^								*P*-value
	FR_0_S_18_FM_0_	FR_0_S_18_FM_1_	FR_0_S_36_FM_0_	FR_0_S_36_FM_1_	FR_1_S_18_FM_0_	FR_1_S_18_FM_1_	FR_1_S_36_FM_0_	FR_1_S_36_FM_1_	
Reproductive performance									
Initial weight, g/♀	200.5	203.25	201.75	203.5	202.25	201.5	202.5	201.5	0.001
Time to 1 st spawning (day)	23.75^c^	19.75^d^	45.50^a^	37.75^b^	18.50^d^	15.50^e^	21.25^cd^	20.50 ^d^	0.001
Inter-spawning intervals (days)	16.75^cd^	14.75^d^	25.50^a^	22.75^b^	14.75^d^	10.50^e^	17.75^c^	15.00^d^	0.001
Number of spawning ♀/hapa	10.25^b^	9.75^b^	5.50^c^	6.50^c^	12.00^b^	18.00^a^	9.75^b^	12.00^b^	0.001
Average number of spawned ♀/hapa	31.25^b^	28.75^b^	15.50^d^	24.25^c^	37.00^a^	37.25^a^	28.75^b^	31.50^b^	0.001
Total number of spawnings per hapa	320.50^cd^	281.00^c^	85.50^e^	157.25^e^	443.25^b^	671.25^a^	281.00^c^	378.75^bc^	0.001
Average number of spawned ♀ per treatment	7.122^cd^	6.244^d^	1.900^e^	3.494^e^	9.850^b^	14.917^a^	6.244^d^	8.417^bc^	0.001
Average number of eggs per each spawning ♀	430.0^bc^	482.5^abc^	397.5^c^	410.0^c^	507.5^ab^	527.5^a^	482.5^abc^	462.5^abc^	0.001
Total number of eggs/hapa	95418.3^d^	87117.9^d^	11956.7^e^	34326.4^e^	184965.3^b^	294624.4^a^	87117.9^d^	122650.2^cd^	0.001
Average absolute fecundity (number of eggs/♀)	2120.4^d^	1936.0^d^	265.7^e^	762.8^e^	4110.3^b^	6547.2^a^	1936.0^d^	2725.6^cd^	0.001
Relative fecundity (eggs number per 1 g of ♀ body weight)	8.12^d^	7.10^d^	0.97^e^	2.81^e^	15.68^b^	24.41^a^	7.10^d^	10.08^cd^	0.001
Hatching performance, egg weight and larval length									
Days to hatch	5.25^b^	5.75^ab^	3.25^c^	4.75^b^	5.75^ab^	6.50^a^	4.75^b^	5.75^ab^	0.001
Sperm count ♂ (*10^6^/mL)	171.25^b^	198.00^a^	117.25^c^	151.75^b^	208.50^a^	217.50^a^	197.00^a^	204.25^a^	0.001
Dead sperm (%)	17.00^bc^	13.75^cd^	28.25^a^	19.75^b^	11.25^d^	11.00^d^	12.75^cd^	10.75^d^	0.001
Hatchability (%)	71.25^cd^	78.50^bc^	60.00^e^	69.50^d^	81.75^ab^	88.75^a^	73.50^cd^	78.75^bc^	0.001
Yolk-sac absorption (days)	4.75^b^	6.25^a^	3.00^c^	4.75^b^	5.75^ab^	6.25^a^	6.00^a^	5.25^ab^	0.001
Egg weight (mg)	4.983^cd^	5.156^cd^	4.751^d^	4.958^cd^	6.105^ab^	6.458^a^	5.643^bc^	5.493^bcd^	0.001
Egg diameter (mm)	2.14	2.145	2.133	2.14	2.135	2.14	2.135	2.148	0.677
Number of fry/hapa	68104.5^de^	127922.3^bc^	7059.3^f^	23849.3^ef^	151210.3^b^	261791.3^a^	63993.8^de^	96557.5^cd^	0.001
Larval length (mm)	8.55^c^	9.30^c^	6.81^e^	7.64^d^	11.22^a^	11.38^a^	10.76^ab^	10.11^b^	0.001
Larval chemical composition									
Moisture (%)	58.22^ab^	60.01^a^	59.37^ab^	59.34^ab^	59.29^ab^	59.68^a^	57.67^b^	58.46^ab^	0.007
Dry matter (%)	41.78^ab^	39.99^b^	40.63^ab^	40.66^ab^	40.71^ab^	40.32^b^	42.33^a^	41.54^ab^	0.007
Crude protein (%)	61.91^cd^	64.11^ab^	58.24^e^	62.35^bcd^	63.62^abc^	64.59^a^	61.26^d^	62.85^abcd^	0.001
Crude lipid (%)	31.74	31.97	33.86	32.95	32.58	31.65	32.67	31.97	0.057
Ash (%)	2.37	3.04	3.66	3.54	3.05	3.05	2.97	2.8	0.129

1 Treatments: FR0S18FM0 = ponds with no fertilizer (FR) at salinity (S) 18ppt and fish fed diet without fishmeal (FM); FR0S18FM1 = nonfertilized ponds at salinity 18ppt and fish fed diet with FM; FR0S36FM0 = nonfertilized ponds at salinity 36ppt and fish fed diet without FM; FR0S36FM1 = nonfertilized ponds at salinity 36ppt and fish fed diet with FM; FR1S18FM0 = fertilized ponds at salinity 18ppt and fish fed diet without FM; FR1S18FM1 = fertilized ponds at salinity 18ppt and fish fed diet with FM; FR1S36FM0 = fertilized ponds at salinity 36ppt and fish fed diet without FM, and FR1S36FM1 = fertilized ponds at salinity 36ppt and fish fed diet with FM; FS + BVL = fish sludge + Beta vulgaris leaves

**Table 17:** Results of three-way ANOVA analysis with SPSS for reproductive fertility, hatchability, egg weight, larval length and larval chemical composition in red tilapia broodstock (♀ and ♂) tested with different levels of FS + BVL fertilizer (FR), salinity (S), and fishmeal-enriched (FM_1_)/fishmeal-free (FM_0_) diets

Dependent Variable	*P*-value of the main effects			*P*-value of the interaction effects			
	fertilizer effect	Salinity effect	Fishmeal effect	FR*S	FR*FM	S*FM	FR*S*FM
Initial weight, g/♀	0.591	0.453	0.243	0.591	0.012	0.591	0.747
Time to 1 st spawning (day)	0.000	0.000	0.000	0.000	0.002	0.641	0.462
Inter-spawning intervals (days)	0.000	0.000	0.000	0.000	0.001	0.012	0.189
Number of spawning ♀/hapa	1.000	0.777	0.098	0.263	0.777	0.572	0.777
Average number of spawned ♀/hapa	0.000	0.001	0.227	0.044	0.709	0.327	0.266
Total number of spawnings per hapa	0.000	0.000	0.000	0.000	0.000	0.446	0.005
Average number of spawned ♀ per treatment	0.000	0.000	0.000	0.000	0.138	0.613	0.138
Average number of eggs per each spawning ♀	0.000	0.000	0.000	0.183	0.004	0.008	0.106
Total number of eggs/hapa	0.000	0.000	0.000	0.000	0.001	0.013	0.452
Average absolute fecundity (total number of eggs/♀)	0.000	0.000	0.000	0.391	0.046	0.016	0.200
Relative fecundity (eggs number per 1 g of ♀ body weight)	0.000	0.000	0.000	0.391	0.046	0.016	0.200
Initial weight, g/♀	0.000	0.003	0.292	0.831	0.292	0.194	0.966
Time to 1 st spawning (day)	0.000	0.000	0.000	0.114	0.129	0.002	0.429
Inter-spawning intervals (days)	0.000	0.000	0.000	0.114	0.129	0.002	0.429
Number of spawning ♀/hapa	0.000	0.000	0.000	0.074	0.108	0.004	0.398
Hatching performance, egg weight and larval length							
Days to hatch	0.000	0.000	0.000	0.096	0.732	0.096	0.309
Sperm count ♂ (*10^6^/mL)	0.000	0.000	0.000	0.676	0.350	0.917	0.406
Dead sperm (%)	0.000	0.000	0.000	0.002	0.000	0.187	0.053
Hatchability (%)	0.000	0.000	0.000	0.007	0.033	0.000	0.247
Yolk-sac absorption (days)	0.000	0.000	0.025	0.001	0.000	0.119	0.061
Body composition of fish larvae							
Moisture (%)	0.131	0.057	0.020	0.010	0.620	0.240	0.071
Dry matter (%)	0.131	0.057	0.020	0.010	0.622	0.240	0.071
Crude protein (%)	0.000	0.000	0.000	0.299	0.006	0.052	0.309
Crude lipid (%)	0.231	0.016	0.098	0.057	0.482	0.508	0.318
Ash (%)	0.389	0.097	0.658	0.020	0.403	0.263	0.471

FR*S = interaction of fertilizer and salinity; FR*FM = interaction of fertilizer and fishmeal; S*FM = interaction of salinity and fishmeal; FR*S*FM = interaction of fertilizer, salinity and fishmeal; FS + BVL = fish sludge + Beta vulgaris leaves

Significant differences were found in larval moisture, dry matter, and crude protein, while lipid and ash showed no significant variation among treatments. The highest crude protein levels were recorded in groups FR_1_S_18_FM_1_, FR_0_S_18_FM_1_, FR_1_S_18_FM_0_, and FR_1_S_36_FM_1_, with no significant differences among them. Overall, the main effects of fertilization, salinity, and diet were stronger than their interaction effects.

## Discussion

This study evaluated the influence of FS + BVL fertilizer on soil properties in low-silt ponds and its implications for the health, physiology, and reproductive performance of red tilapia (*Oreochromis* sp.) under saline conditions and plant-based diets. The findings highlight the vital role of FS + BVL compost in improving water quality, soil fertility, growth and somatic indices, physiological responses, reproductive performance, carcass composition and soil properties, contributing to sustainable aquaculture practices in marginal environments.

### Water quality

In saline water environments, the use of organic fertilizers can be particularly advantageous because saline water tends to limit nutrient availability and the biological productivity of ponds due to osmotic stress on both aquatic plants and fish [66, 67]. Organic fertilizers can help mitigate these effects by supplying readily available nutrients that enhance primary productivity, thus fostering an environment more conducive to fish growth [16]. In addition, organic fertilizers can improve the structure of low-fertility soils by enhancing their water-holding capacity and reducing the effect of high salinity on pond chemistry [68, 69]. Moreover, the breakdown of organic fertilizers releases humic substances, which can bind with salt ions, reducing their free concentration in the water and mitigating the osmotic pressure on fish [70, 71]. This can result in improved performance, productivity, and fry survival rates of fish species sensitive to salinity fluctuations, such as red tilapia. In line with previous literature, the results of the current study found that ponds fertilized with FS + BVL under both salinities recorded higher levels of organic matter, total and organic carbon, total and available nitrogen, as well as total and available phosphorus.

The present study revealed considerable improvements in water quality in fish ponds fertilized with FS + BVL with higher levels of DO, chlorophyll a, and zooplankton; lower TAN and NO_2_ levels; and relatively stable pH under varying salinities (18 and 36‰) and diets (FM_0_ and FM_1_). These findings are consistent with those of Sallam et al. [16], who documented similar improvements in the mentioned water quality parameters in Nile tilapia tanks when treated with different organic fertilizers. The increase in DO can be attributed to higher levels of chlorophyll, where photosynthesis occurs and oxygen is produced in the ponds, while the decline in TAN and NO_2_ can be attributed to the increased numbers of zooplankton, which contain different types of microbes that break down these toxic compounds [72, 73]. pH levels were also higher in the fertilized groups, building more alkaline conditions, indicating that treatments delivered an optimal pH environment for red tilapia. This result concurs with Khanjani et al. [74], who noticed that pH stability is linked to microbial activity, as in biofloc systems. A constant pH framework is substantial for nutrient solubility and availability, and unbalanced pH levels can obstruct aqueous ecosystems [75]. The fertilized groups recorded higher zooplankton numbers compared to the non-fertilized, which is associated with lower nitrogen by-products. This may be due to organic fertilizers containing a microbial community [72, 76] capable of degrading these harmful products through immobilization and nitrification processes, improving nitrogen cycling, and promoting the ecosystem. Moreover, fish sediment may also contain some microbes with probiotic characteristics such as *Bacillus*, *Lactobacillus brevis*, *Lactobacillus coryniformis*, *Lactobacillus collinoides*, and *Lactobacillus farciminis* [77].

### Soil properties and fertilization effects

Soil quality and nutrient content play an important role in the abundance and prosperity of natural food chain, including phyto- and zooplankton in aquaculture ponds. Prior research has demonstrated that fertilizer significantly enhances soil quality and, consequently, natural food availability. Research studies have examined the impact of soil quality and fertilization strategies on improving survival, growth, and productivity in fish ponds [78]. Nevertheless, more detailed research focusing on specific reproductive indicators like fecundity and egg development is needed to understand the effects of soil quality and fertilization strategies [79]. The findings of the present study demonstrate that fertilized ponds had superior reproductive outcomes in comparison to non-fertilized ponds, aligning with higher concentrations of both chlorophyll a and zooplankton numbers. This may be due to the fact that fertilized ponds, regardless of soil type and culture system, yielded greater live natural food compared to unfertilized ponds [78]. Lomartire et al. [80] affirmed that the abundance and diversity of plankton populations strategically influence a fish’s spawning behavior and success. A reliable and varied food supply serves as a crucial environmental signal for fish to commence spawning. In this context, biofloc technology (BFT) generally improves tilapia reproductive performance by enhancing water quality and providing supplementary nutrition, resulting in enhanced fecundity and offspring survival due to the nutrient-rich environment created by bioflocs [81]. This microbial biomass is rich in essential fatty acids, protein, and a variety of vitamins and minerals, including phosphorus. Broodstock health, reproductive performance, and egg quality can be enhanced by consuming biofloc [82].

### Growth, carcass composition, and somatic indices

The investigation revealed that the use of FS + BVL fertilizer had no notable effect on the growth or feed efficiency of red tilapia broodstock (♀) across various salinity and diet conditions, likely because energy was focused on reproduction. However, fertilization enhanced carcass characteristics and increased crude protein and ash content in fish on a plant-based diet (FM0) at both 18 and 36‰ salinity levels, making their composition comparable to that of fish on a fishmeal diet (FM_1_). The elevated protein content in fish muscles can be attributed to beetroot’s role in promoting digestion by improving stomach and intestinal functions, acting as an anti-inflammatory, and improving appetite [31, 33], thus maximizing protein absorption. The higher ash content is due to the fertilizer containing beetroot leaves, which are rich in minerals [32, 33]. Additionally, crude lipid content was significantly lower in the fertilized (FS + BVL) groups compared with the non-fertilized groups in both salinities and diet types. Higher crude protein and ash contents reflect improved nutrient density and better protein utilization [83, 84], while lower crude fat indicates a slighter carcass with a more proportional nutritional profile [85] in the FS + BVL-treated groups. Regarding organ indices, the improvement in reproductive organs (GSI and TSI) in fish raised in FS + BVL fertilized groups can be attributed to the fact that organic fertilization is known to improve metabolic pathways and provide biostimulants to enhance metabolite synthesis [86]. Moreover, organic fertilizers are a naturally available mineral source that enhances secondary production in fish ponds by stimulating heterotrophic bacteria and enriching soil fertility [87].

### Physiological responses

#### Serum screening

Serum screening of red tilapia broodstock in the present study shows that the combination of FS and BVL gives significantly better results in fish well-being and welfare in low-silt ponds, which is reflected in their reproductive performance. The existing results emphasize the notable enhancements in serum biochemical markers of red tilapia raised in fertilized (FS + BVL) groups under both salinities (18 and 36‰) and diets (FM_0_ and FM_1_) compared with those reared in non-fertilized treatments. The application of fertilizer resulted in significant decreases in liver enzymes (AST and ALT), kidney metabolic functions (urea, uric acid, and creatinine), stress hormone (cortisol), and serum cholesterol levels in both sexes (♀ and ♂) under both 18 and 36‰ salinity and FM_0_ and FM_1_ diets, indicating enhanced lipid metabolism, liver and kidney functions, and reduced hepatic stress. These findings align with those of Sallam et al. [16] and Naiel et al. [76]. Cholesterol is an important metabolite for assessing the nutritional status of fish and is affected by the protein source they consume [88, 89]. Lower levels of urea, uric acid, and creatinine indicate more efficacious protein metabolism, improving water quality and lessening stress on the filtration system, as observed by Naiel et al. [76] and Sallam et al. [16]. Diminished AST and ALT levels suggest improved utilization of dietary protein for body protein synthesis [73], while decreased cortisol levels demonstrate decreased stress levels [90]. These outcomes underscore the prospect of using FS + BVL fertilizer to improve the welfare and health status of tilapia fish when low-silt ponds are used in aquaculture or when salinity fluctuates.

#### Antioxidant capacity and immune response

The study underlines the efficacy of FS + BVL fertilizer in improving immune responses and antioxidant capacity of red tilapia broodstock reared in low-silt ponds under the effect of saline water and plant-based diets. Using the FS + BVL fertilizer considerably increased GP_x_, CAT, and SOD activities and significantly decreased MDA levels in both ♀ and ♂ in all fertilized groups, demonstrating enhanced oxidative stress management under different salinities (18 and 36‰) and diets (FM_0_ and FM_1_), which is consistent with the results of Sallam et al. [16]. Compared with those in the fertilized groups, fish (♀& ♂) reared in non-fertilized ponds showed lower GP_x_, CAT, and SOD activities and increased MDA levels, except for the FR_0_S_18_FM_1_ group, highlighting the interactive effect of salinity level and diet type. Additionally, augmented lysozyme and TP activity in the FS + BVL groups implies a stronger innate immune defense, which is paramount for fish well-being and resilience. The advantageous outputs correlated to the FS + BVL fertilizer can be assigned to the beneficial role in improving pond soil fertility and water quality and providing an additional food web consisting of phyto- and zooplankton. BVL contains bioactive substances such as betalains, flavonoids, phenolic acids, carotenoids, minerals, and vitamins [32, 33] that can promote the immune system and antioxidant defense. Furthermore, BVL is rich in beta-carotene, which provides an excellent source of powerful antioxidants, aids in blood production, and increases red blood cell count [31, 32]. The higher globulin levels in the FR_1_S_18_FM_1_ (♀& ♂) group and FR_1_S_18_FM_0_ (♀) group compared to the other groups tested reflect the profound interaction effect between fertilizer, salinity, and diet. Organic manure, especially FS + BVL, from aquaculture byproducts and agricultural manure improves the physiological and immunological health of red tilapia broodstock reared in low-silt ponds at varied salinities.

#### Digestive enzymes

This study examined how FS + BVL fertilizer in low-silt ponds affected red tilapia broodstock digestive enzymes (protease, lipase, and amylase). All FS + BVL-fertilized fish showed increased enzyme activity across salinity and diet types, indicating improved protein digestibility. Under each salinity class, fish groups fed a fishmeal-enriched diet (FM_1_) across both fertilized and non-fertilized ponds exhibited enhanced digestive enzyme activity, compared to those fed a plant-based diet (FM_0_). The synergistic effect of fertilizer, diet and salinity on digestive enzymes resulted in the highest levels in fish (♂ and ♀) reared in the FR_1_S_18_FM_0_ and FR_1_S_18_FM_1_ groups and the lowest values in the FR_0_S_36_FM_0_ and FR_0_S_36_FM_1_ groups, while the activity in the FR_1_S_36_FM_0_ group was improved to be comparable to the FR_0_S_18_FM_0_ group. These results align with those of Sallam et al. [16], who documented promoted digestive enzyme activity in Nile tilapia treated with various organic fertilizers. Similarly, adding *Moringa oleifera* leaf extract to the diet of red tilapia enhanced the activity of these digestive enzymes under high salinity of 32‰ [91]. The improved protease activity in the fertilized groups is probably a result of the advantageous impacts of FS + BVL on gut health and enzyme production. Similarly, elevated amylase levels may be a consequence of raised availability of carbohydrate content and enhanced gut health, leading to enhanced carbohydrate assimilation and energy utilization. The notably higher lipase activity in the FS + BVL groups reflects better fat assimilation, supporting enhanced energy utilization. Moreover, BVL is known to act as an anti-inflammatory, enhance appetite, promote digestion, regulate bowel activity, and improve stomach and intestinal functions [31, 33]. In addition, its content of minerals and vitamins [32, 33], along with beta-carotene, which helps in blood production, may boost the gastrointestinal immunity and feed digestibility [31, 32].

### Sexual steroids and reproductive performance

As salinity increases above 18‰, reproductive performance and fry production per unit female weight are impaired, indicating a potential struggle to balance energy allowance to hatching and osmoregulation in red tilapia [21]. Notably, the current examination documents that the FS + BVL application positively affects the reproductive performance of red tilapia, improving both sexual reproductive hormones and offspring production in all FS + BVL-treated groups. Progesterone (♀) and testosterone (♂) levels showed significant improvement in fish cultured in the FS + BVL fertilized groups under both salinities (18 and 36‰) and diets (FM_0_ and FM_1_) compared to those grown in the unfertilized conditions. Sexual steroids play a vital role in multiple basic physiological functions in all vertebrates, and estimating their blood levels seems to be a useful tool for evaluating the reproductive cycle of fish [92, 93]. Testosterone is produced by Leydig cells, which play a prominent role in spermatogenesis and the evolution of secondary sexual traits in ♂ [94, 95]. Progestins are pivotal regulators of reproduction in fish and key intermediates in the biosynthesis of numerous functional steroids in fish, including androgens such as estradiol-17a, estradiol-17b, and 20b-dihydroxyprogesterone [96, 97].

Applying FS + BVL fertilization significantly enhanced reproductive traits in red tilapia, particularly female fecundity, spawning frequency, and egg production. Improvements were noted in sperm count and viability, egg hatchability, and larval length in all treated groups compared to controls. Additionally, the fertilizer improved fry carcass quality, increasing dry matter and crude protein content in the treated groups. To our knowledge, no previous studies have directly examined the effect of organic fertilization on tilapia reproduction under salinity stress, especially in low-silt pond soils. Similar improved findings have been documented with red tilapia broodstock under high salinity conditions, including those reared in a biofloc system [98] and those fed nanocurcumin supplements [22] or *Moringa oleifera* leaf extract [91]. The authors attributed these improvements in fertility and reproductive traits of maternal red tilapia to the role of biofloc, nanocurcumin, and *Moringa oleifera* supplements in providing the fish with good sources of diets that ensure sufficient energy for osmoregulation and reproduction at the same time.

The application of FS + BVL fertilizer positively influences gonadal development, hormonal profiles, and reproductive traits in red tilapia, enhancing their fertility. This fertilizer fosters a nutrient-rich environment that counters the adverse effects of high salinity on tilapia reproduction. Additionally, it improves soil properties and increases phytoplankton and zooplankton availability, which are vital secondary food sources that provide essential energy for osmoregulation and reproduction in fish. This is in line with Volkoff and London [99], who reported that the nutritional quality of maternal fish greatly impacts egg quality, successive embryonic evolution, and larval development. The nutrient reserve in the maternal stock plays a crucial role in meeting the metabolic prerequisites of generating embryos [100, 101]. The interaction between FS + BVL fertilizer and salinity significantly impacts stress stimulators, proposing its potential to modify physiological responses and inhibit stress indicators in fish under different salinity conditions. Moreover, the suggested mechanism implicates the FS + BVL fertilizer’s ability to improve reproductive function in fish stems from amplifying immune defense, antioxidant, and anti-inflammatory characteristics [32, 33]. The abundance of bioactive components such as betalains, flavonoids, minerals, vitamins, and beta-carotenes may lessen oxidative stress in gametes, lower inflammation in reproductive organs, and modulate sex steroids, leading to amplified sperm and egg quality and overall reproductive traits. Furthermore, BVL’s effect on blood production [31, 32] may enhance the delivery of nutrients and oxygen to the reproductive organs, further helping to achieve superior reproductive outputs in red tilapia [22].

## Conclusion

The study demonstrated that applying FS + BVL organic fertilizer in low-silt ponds under 18 and 36 ppt salinity significantly improved the overall culture environment and reproductive performance of red tilapia broodstock fed both FM_1_ and FM_0_ diets. The fertilizer enhanced soil fertility (carbon, nitrogen, phosphorus), water quality (higher dissolved oxygen, chlorophyll *a*, zooplankton; lower ammonia and nitrite), and consequently improved fish carcass composition (notably higher crude protein).

Physiologically, FS + BVL application improved gonadal development (higher GSI and TSI), serum biochemical health, antioxidant and immune responses, and digestive enzyme activity, reflecting reduced stress and improved metabolism. Reproductive outcomes—including sex hormone levels, fecundity, sperm vitality, hatchability, fry yield, and larval quality, were all markedly enhanced, even under high salinity and plant-based diets. Overall, FS + BVL acts as a sustainable biofertilizer that boosts pond productivity, fish health, and reproduction in saline and nutrient-poor environments, promoting eco-friendly aquaculture expansion. Future research should elucidate the molecular mechanisms governing these fertility and metabolic improvements.

## Data Availability

Data available upon request from the corresponding author.

## Abbreviations

FS: Fish sludge
BVL: Beta vulgaris leaves
FS + BVL: Fish sludge + compost of Beta vulgaris leaves
FR: Fertilizer
FR_0_: Unfertilized group
FR_1_: Fertilized group
S: Salinity
M_0_: Fishmeal-free diet/or plant-based
M_1_: Fishmeal-enriched diet
IBW: Initial body weight
FBW: Final body weight
WG: Weight gain
ADG: Average daily gain
K: Condition factor
HSI: Hepatosomatic
VSI: Viscerosomatic Index
TSI: Testosomatic Index
GSI: Gonadosomatic Index
FCR: Feed conversion ratio
TAN: Total ammonia nitrogen
NO_2_: Nitrite
NH_3_: Non-ionized ammonia
FR_0_S_18_FM_0_: Unfertilized fish group at 18‰ salinity and fishmeal-free diet
FR_0_S_18_FM_1_: Unfertilized fish group at 18‰ salinity and fishmeal-enriched diet
FR_0_S_36_FM_0_: Unfertilized fish group at 36‰ salinity and fishmeal-free diet
FR_0_S_36_FM_1_: Unfertilized fish group at 36‰ salinity and fishmeal- enriched diet
FR_1_S_18_FM_0_: Fertilized fish group at 18‰ salinity and fishmeal-free diet
FR_1_S_18_FM_1_: Fertilized fish group at 18‰ salinity and fishmeal- enriched diet
FR_1_S_36_FM_0_: Fertilized fish group at 36‰ salinity and fishmeal- free diet
FR_1_S_36_FM_1_: Fertilized fish group at 36‰ salinity and fishmeal- enriched diet
SGR: Specific growth rate
ADG: Average daily gain
TP: Total protein
AST: Aspartate aminotransferase
ALT: Alanine aminotransferase
CAT: Catalase
SOD: Superoxide dismutase
MDA: Malondialdehyde
GPx: Glutathione peroxidase
DO: Dissolved oxygen
